# Circular RNA: A potential diagnostic, prognostic, and therapeutic biomarker for human triple-negative breast cancer

**DOI:** 10.1016/j.omtn.2021.06.017

**Published:** 2021-07-02

**Authors:** Tian Tian, Yangzhi Zhao, Jingying Zheng, Shunzi Jin, Zhongshan Liu, Tiejun Wang

**Affiliations:** 1 Department of Radiation Oncology, The Second Affiliated Hospital of Jilin University, Changchun 130041, China; 2 Department of Hematology, The First Hospital of Jilin University, Changchun 130021, China; 3 Department of Gynecology and Obstetrics, The Second Affiliated Hospital of Jilin University, Changchun 130041, China; 4 NHC Key Laboratory of Radiobiology, Jilin University, Changchun 130021, China

**Keywords:** circular RNAs, triple-negative breast cancer, biomarker, diagnosis, prognosis, therapeutic potential

## Abstract

Triple-negative breast cancer (TNBC), which is the most malignant subtype of breast cancer (BC), accounts for 10%–20% of all BC cases. TNBC, which occurs more frequently in young women, is characterized by high rates of cell proliferation and metastasis and poor prognosis. Chemotherapy is the primary systemic therapeutic strategy for TNBC. However, chemotherapy is largely unsuccessful, and effective targeted therapies for TNBC have not been established. Therefore, it is a matter of great urgency to identify precise molecular targets for the promising prognosis of patients with TNBC. Circular RNAs (circRNAs), which are a type of non-coding RNAs (ncRNAs), are abundantly expressed in the eukaryotic cells and exhibit diverse cellular functions. The roles of circRNAs are to sponge microRNA or RNA-binding proteins, regulate gene expression, and serve as templates for translation. Here, we review the current findings on the potential of circRNAs as a diagnostic, prognostic, and therapeutic biomarker for TNBC. However, further studies are essential to elucidate the functions of circRNAs in TNBC. This review also discusses the current limitations and future directions of TNBC-associated circRNAs, which can facilitate the translation of experimental research into clinical application.

## Graphical abstract

**Figure:**
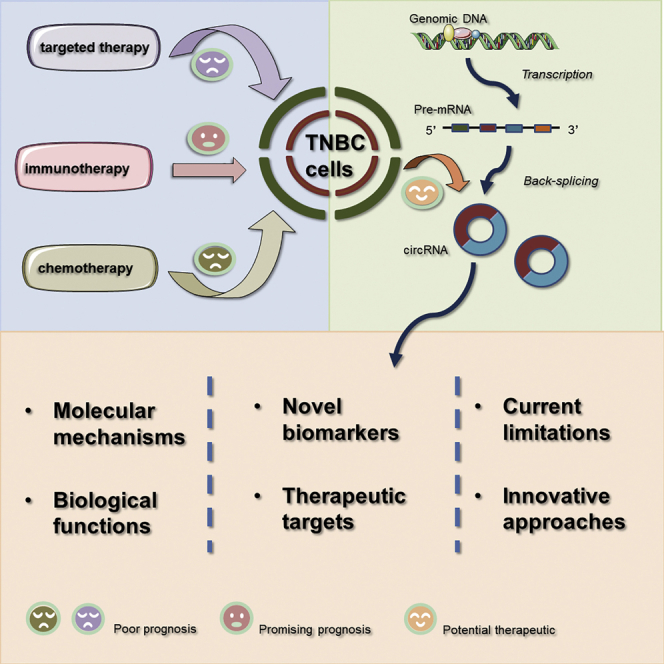

## Teaser

In this manuscript, the efficacy of current therapeutic strategies was addressed for human triple-negative breast cancer (TNBC). Then, dysregulated circular RNAs (circRNAs) were summarized to reveal the biomarker potential and therapeutic mechanism. Finally, novel perspectives were provided for future studies to improve the poor overall survival of TNBC.

## Introduction

Breast cancer (BC) is a threat to human health, as it is associated with a high mortality rate. According to the American Cancer Society report of 2019, 271,000 new BC cases and 42,260 BC-related deaths were reported in the United States.^1^ Among the BC subtypes, triple-negative breast cancer (TNBC) is associated with poor overall survival (OS) and disease-free survival (DFS).^2^

TNBC, which accounts for 10%–20% of all BC cases, does not exhibit the expression of hormone receptors (estrogen receptor [ER] and progesterone receptor [PR]) and human epidermal growth factor receptor 2 (HER2).^3^^,^^4^ Lehmann et al.^5^ defined TNBC molecular subtypes into four tumor-specific subtypes: basal-like 1 (BL1) and 2 (BL2), mesenchymal (M), and one luminal androgen receptor group (LAR). The phenotypes of TNBC, which is frequent among young patients, include large size and advanced tumor grade.^3^^,^^6^^,^^7^ Patients with TNBC exhibit poor survival rates owing to the metastatic properties of the tumor. TNBCs would have metastasized to local and distant lymph nodes at diagnosis and exhibit high proliferative rates, which may be attributed to genetic and molecular aberrations.^6^^,^^8^, ^9^, ^10^ Women with TNBC, especially African American women, exhibit high rates of early distant recurrence and a poor 5-year prognosis.^6^

The carcinogenesis of TNBC is a complex process that involves genetic mutations and dysregulation of epigenetic pathways. Previous studies have examined the epigenetic changes (such as DNA methylation^11^^,^^12^), non-coding RNA (ncRNA) profile,^13^^,^^14^ and somatic mutation profile in TNBC.^15^^,^^16^ ncRNA LOC339535 (commonly referred to as LINK-A), which is involved in the carcinogenesis of TNBC, is associated with poor prognosis and progression-free survival in patients with TNBC.^17^ Additionally, the dysregulation of various ncRNAs, including microRNAs and long-ncRNAs (lncRNAs), contributes to tumorigenesis and tumor progression.^13^^,^^14^ Thus, the therapeutic potential of targeting ncRNAs has piqued the interest of the scientific community.

Currently, the standard treatment for patients with TNBC is the application of chemotherapeutic agents, such as anthracyclines, alkylating agents, taxanes, or platinum salts.^18^^,^^19^ Poly(ADP-ribose) polymerase (PARP) inhibitors have been approved for the treatment of BRCA mutant TNBC.^20^ Additionally, immune modulators are promising therapeutics for TNBC.^21^ The combination of nab-paclitaxel and atezolizumab (anti-PD-L1) was recently approved by the US Food and Drug Administration (FDA) for the patients with unresectable locally advanced or metastatic TNBC whose tumors express PD-L1 based on a PFS benefit over chemotherapy in the Impassion130 trial.^22^ However, further studies are needed to develop effective targeted therapeutics for TNBC. Recent studies have reported that chemotherapy is associated with unsatisfactory clinical outcomes in patients with TNBC.^18^ Hence, there is a need to identify novel biomarkers, as well as novel genetic and epigenetic therapeutic targets, for TNBC.

The identification of ncRNAs has increased our understanding of the pathogenesis of TNBC and can aid in the development of novel therapeutic strategies for TNBC. More than 80% of cancer-associated single-nucleotide polymorphisms are reported in non-coding regions of the genome. Thus, ncRNAs, which contribute to TNBC carcinogenesis, can be a potential therapeutic target for TNBC.^23^ Circular RNAs (circRNAs), a subclass of ncRNAs abundantly expressed in the eukaryotic cells, are covalent single-chain closed-loop structures that lack terminal 5¢ and 3¢ ends.^24^ Sanger et al.^25^ first discovered circRNAs in 1976. The functions of circRNAs were recently elucidated after the development of sequencing techniques.^24^ Structural characteristics of circRNAs have a decisive effect on their own performances, which means that they have obvious advantages as biomarkers of clinical diseases on account of the high stability and evolutionary conservation.^26^ Hence, further studies are needed to elucidate the role of circRNAs in the regulation of various pathological conditions.^27^ Recently, the regulatory function of circRNAs has been demonstrated in several solid tumors, such as colorectal cancer (CRC) or prostate cancer.^28^, ^29^, ^30^ Additionally, circRNAs are involved in TNBC cell proliferation, apoptosis, migration, and invasion. This review summarizes the role of circRNAs in TNBC and the potential underlying molecular mechanisms to aid therapeutic applications. A comprehensive understanding of circRNAs may provide useful information for the clinical application of circRNA-based biomarkers.

## Efficacy of the current therapeutic strategies for TNBC

### Efficacy of chemotherapy

Chemotherapy is the primary systemic treatment for both early-stage and advanced TNBC, a pathological condition for which molecular targeted therapy has not been developed.^18^^,^^31^ Although TNBC is associated with a high metastatic rate (30%–40%),^32^ neoadjuvant chemotherapy (NAC) and standard surgery are the most effective approaches to improve OS of patients with early-stage TNBC.^33^^,^^34^ Interestingly, even with what is deemed as a poor OS, subsets of patients with TNBC have a better response to chemotherapy than those with other subtypes. It is the so-called TNBC paradox, because patients with TNBC are associated with a high risk of recurrence but also benefit from treatment.^35^ Disease recurrence in patients with TNBC can be attributed to the residual tumor cells that are not killed after NAC.^32^

A recent study^36^ reported that the efficacy of NAC can be enhanced with the use of dose-dense and high-dose regimens in TNBC. Taxanes and anthracyclines are the major therapeutics used in NAC.^37^^,^^38^ Additionally, the correlation of NAC, which is associated with toxicity, with improved pathological complete response rate and event-free survival has not been established. Hence, the application of platinum-based agents to standard NAC has been disputed in clinical practice.^18^

The 5-year survival rate in women with metastatic BC is less than 30%, and almost all patients with metastatic TNBC will ultimately die of the disease.^39^ The European Society for Medical Oncology (ESMO)^40^ and American Society of Clinical Oncology (ASCO)^41^ recommend sequential single-component chemotherapy for patients with metastatic tumors unless the progression of the disease or visceral crisis is rapid. The efficacy of single-component therapeutic agents against metastatic tumors is lower than that of combination therapies. However, combination therapy is associated with high cost and increased toxicity and provides limited survival benefits.^42^

### Efficacy of targeted therapy

Based on the results of clinical or preclinical trials (Table 1), various molecular targets have been proposed to improve the prognosis of TNBC and overcome the intrinsic resistance to chemotherapy. The clinical trials of therapeutic agents, such as PARP inhibitors, phosphoinositide 3-kinase (PI3K) inhibitors, MEK inhibitors, and histone deacetylase (HDAC) inhibitors targeting various signaling pathways,^43^ have yielded beneficial clinical outcomes in approximately 90% of TNBC cases.

**Table 1:** Progressive clinical trials on TNBC target therapy in the search of therapeutic biomarkers

Clinical trial	Phase	Interventions	Indications	Therapeutic target
NCT02163694	III	experimental group: veliparib (o.a.), days 2 to 5 of a 21-day cycle; carboplatin (i.v.), day 1 of a 21-day cycle; paclitaxel (Taxol) (i.v.), days 1, 8, and 15 of a 21-day cycle; control group: placebo (o.a.), days 2 to 5 of a 21-day cycle; carboplatin (i.v.), day 1 of a 21-day cycle; paclitaxel (Taxol) (i.v.), days 1, 8, and 15 of a 21-day cycle	metastatic TNBC	PARP inhibitor
NCT02000622	III	group 1: olaparib (300 mg; o.a.), twice daily at 12-h intervals; group 2: capecitabine (Xeloda) (2,500 mg/m^2^; i.v.), days 1–14, q21; vinorelbine (30 mg/m^2^; o.a.), days 1,8, and q21; or eribulin (1.4 mg/m^2^; i.v.), days 1, 8, and q21	metastatic breast cancer	PARP inhibitor
NCT02032823	III	group 1: olaparib (300 mg; o.a.), twice daily at 12-h intervals; group 2: placebo (300 mg; o.a.), twice daily at 12-h intervals	early-stage TNBC	PARP inhibitor
NCT01945775	III	experimental group: talazoparib (1.0 mg; o.a.), once daily for 21 continuous days; control group: capecitabine, eribulin, gemcitabine, or vinorelbine (o.a.), once daily for 21 continuous days		PARP inhibitor
NCT01623349	I	group 1: olaparib (starting dose 50 mg; o.a.), twice daily; BKM120 (starting dose 40 mg; o.a.), once daily; group 2: olaparib (starting dose 100 mg; o.a.), twice daily; BYL719 (starting dose 250 mg; o.a.), once daily	recurrent TNBC	PI3K inhibitor
NCT02000882	II	BKM120 (o.a.), daily; capecitabine (o.a.), twice a day for 2 weeks on/1 week off, ongoing	TNBC with brain metastases	PI3K inhibitor
NCT03243838	I–II	four cycles of docetaxel and apatinib combination, followed by four cycles of epirubicin and cyclophosphamide combination	early-stage TNBC	small inhibitor of EGFR
NCT02623972	II	eribulin (Halaven) (i.v.) for four cycles, followed by AC (i.v.) for four cycles	advanced TNBC	inhibitor of microtubule dynamics
NCT02120469	I	everolimus (Afinitor) (o.a.), daily; eribulin mesylate (Halaven) (i.v.) twice every month	metastatic TNBC	mTOR inhibitor
NCT02672475	I	paclitaxel (Taxol) (i.v.), weekly with 3 weeks on/1 week off; galunisertib (LY2157299) (o.a.), twice daily with 3 weeks on/1 week off	metastatic TNBC	inhibitor of the TGF-β receptor I kinase
NCT02632071	I	ACY-1215 (ricolinostat) (o.a.), daily with 3 weeks on/1 week off; nab-paclitaxel (Abraxane) (i.v.), weekly with 3 weeks on/1 week off	advanced TNBC	HDAC6 blocker
NCT02393794	I–II	romidepsin (Istodax) (i.v.), twice every 3 weeks; cisplatin (Platinol), (i.v.), every 3 weeks	TNBC	HDAC inhibitor
NCT02425891	III	experimental group: MPDL3280a (atezolizumab) (i.v.); nab-paclitaxel (Abraxane) (i.v.); control group: placebo; nab-paclitaxel (Abraxane) (i.v.)	locally advanced or metastatic TNBC	anti-PD-L1
NCT02309177	I	group 1: nab-paclitaxel (i.v.), weekly for 3 weeks every month; nivolumab (Opdivo) (i.v.), every 2 weeks starting at 3 months; group 2: nab-paclitaxel (i.v.), once every 3 weeks; nivolumab (Opdivo) (i.v.), every 3 weeks starting at 3 months	recurrent metastatic TNBC	anti-PD-1
NCT02366949	I	experimental group: BAY1217389 (o.a.), twice daily; paclitaxel (Taxol) (i.v.), weekly; control group: paclitaxel (Taxol) (i.v.), weekly	advanced TNBC	MPS1
NCT02595320	II	group 1: capecitabine (Xeloda) (1,500 mg; o.a.), twice daily with 1 week on/1 week off; group 2: capecitabine (Xeloda) (1,250 mg; o.a.), twice daily with 2 weeks on/1 week off	metastatic TNBC	alkylating agent; tumor-selective and tumor-activated cytotoxic agent
NCT02929576	III	group 1: Xtandi and Taxol, enzalutamide (Xtandi) (o.a.), daily, ongoing; paclitaxel (Taxol) (i.v.), weekly for 16 weeks; group 2: placebo (o.a.), daily, ongoing; paclitaxel (Taxol) (i.v.), weekly for 16 weeks; group 3: Xtandi followed by Taxol, enzalutamide (Xtandi) (o.a.), daily, ongoing; paclitaxel (Taxol) (i.v.), weekly for 16 weeks	advanced TNBC	synthetic non-steroidal antiandrogen
NCT02187991	II	group 1: paclitaxel (Taxol) (i.v.), 3 times a month, ongoing; group 2: paclitaxel (Taxol) (i.v.), 3 times a month, ongoing; alisertib (o.a.), 3 times a week, ongoing	advanced TNBC	aurora A kinase inhibitor
NCT02950064	I	BTP-114 (i.v.), once every 3 weeks, ongoing	advanced TNBC	albumin-binding cisplatin prodrug
NCT02624700	II	pemetrexed (i.v.), every 2 weeks; sorafenib (o.a.), twice daily for 5 days	recurrent or metastatic TNBC	small inhibitor of several tyrosine protein kinases, such as VEGFR, PDGFR, and Raf family kinases (more avidly C-Raf than B-Raf)
NCT02978716	II	group 1 (chemotherapy only): gemcitabine (Gemzar) and carboplatin (Paraplatin) (i.v.) on days 1 and 8, ongoing; group 2 (trilaciclib and chemotherapy): trilaciclib (G1T28) (i.v.) on days 1 and 8, ongoing; gemcitabine (Gemzar) and carboplatin (Paraplatin) (i.v.) on days 1 and 8, ongoing; group 3 (trilaciclib and chemotherapy): trilaciclib (G1T28) (i.v.) on days 1, 2, 8, and 9, ongoing; gemcitabine (Gemzar) and carboplatin (Paraplatin) (i.v.) on days 2 and 9, ongoing	recurrent or metastatic TNBC	CDK4/6 inhibitor

i.v., intravenous; o.a., oral administration; mTOR, mammalian target of rapamycin; HDAC6, histone deacetylase 6; PD-L1, programmed cell death ligand 1; MPS1, serine/threonine kinase monopolar spindle 1; PD-1, programmed cell death receptor; CDK, cyclin-dependent kinase.

PARP is a constitutively expressed nuclear enzyme that activates intracellular signaling pathways by transferring ADP-ribose from NAD+ to target proteins at the sites of DNA damage. Hence, PARP is involved in DNA repair, cellular proliferation, and oncogene regulation.^44^ Various comprehensive reviews^20^^,^^36^^,^^45^^,^^46^ have proposed three pathways through which PARP inhibitors may impact the efficacy of cancer treatment. PARP may function as a radiosensitizer or chemosensitizer, exhibit selective growth-inhibitory activity against BRCA-mutated BC, and leverage putative “BRCA-like” defects and defects in DNA repair. A recent study reported that the lesion size decreased by more than 30% in five out of 10 (50%) patients with BRCA-mutated TNBC with the PARP inhibitor olaparib.^47^

Hyper-activation of the PI3K/AKT pathway, which is caused by oncogenic mutations in TNBC (10.2%),^48^ plays a crucial role in regulating cell growth, metabolism, and survival. Furthermore, what should also be taken into consideration are other mutations reported in this signaling pathway, including phosphatase and tensin homolog loss (9.6%), AKT amplification, and tumor suppressor phosphatase inositol polyphosphate 4-phosphatase type II (INPP4B) loss.^49^, ^50^, ^51^ Several studies are ongoing for evaluating the therapeutic efficacy of PI3K inhibitors or AKT inhibitors (buparlisib or ipatasertib) in patients with solid tumors, including TNBC.^52^

The expression of growth factor receptors, including epidermal growth factor receptor (EGFR), vascular endothelial growth factor receptor (VEGFR), and fibroblast growth factor receptor (FGFR), is upregulated in TNBC. The combination of lapatinib and imatinib is reported to exhibit synergistic growth-inhibitory activity against the TNBC cell line MDAMB-468.^53^

### Effects of immunotherapy

Immunotherapy has gradually become a hotspot in the treatment of malignant tumors in recent years. Immune checkpoint inhibitors (ICIs) block the transmission of immunosuppressive signals by single or combined therapy to reactivate the immune response of T cells and restore immune activity in the tumor microenvironment (TME) and eventually contribute to the anti-tumor effect. Particularly, the monoclonal antibodies (mAbs), targeting programmed cell death protein-1 (PD-1) and its ligand (PD-L1), have attracted much attention. PD-1 (CD279) is located on the PDCD1 gene, and its ligands are PD-L1 (CD274 or B7-H1) and PD-L2 (CD273 or B7-DC).^54^ Compared to other subtypes of BC, genomic instability and high mutation load make TNBC the most immunogenic subtype, with more stromal tumor-infiltrating lymphocytes (TILs) and higher expression of PD-L1.^55^^,^^56^

The pathway of PD-1/PD-L1 plays an important role in the process of cancers evading T cell-mediated tumor-specific and pathogen-specific immunity by inducing T cell tolerance, inhibiting proliferation of T cells, inhibiting secretion of cell factors, and blocking the antigen presentation process.^57^ When PD-1 binds to PD-L1, the phosphorylation is facilitated by tyrosine in immunoreceptor tyrosine-based switch motif (ITSM), a structural component of PD-1, leading to the dephosphorylation of the downstream protein kinases Syk and PI3K. Then, the combination downregulates the activation of downstream pathways to inhibit the transcription and translation of genes and negatively regulate immune responses to cancer in the end.^58^

The KEYNOTE-012 study^59^ reported the safety and efficacy of pembrolizumab monotherapy in the treatment of patients with advanced PD-L1-positive tumors, including TNBC. There were 111 patients with TNBC, and the positive rate of PD-1 was 58.6%, 32 of whom participated in this clinical trial. In terms of safety, common adverse reactions included myalgia (18.8%), fatigue (18.8%), arthralgia (18.8%), and nausea (15.6%), among which 5 patients (15.6%) had grade 3 or more adverse reactions. As for efficacy, among the 27 patients treated with pembrolizumab, the overall response rate (ORR) was 18.5% (95% confidence interval [CI]: 6.3%), the median progression-free survival (mPFS) was 1.9 months (95% CI: 1.3–4.3 months), and the median overall survival (mOS) was 10.2 months (95% CI: 5.3–1.5 months). This study shows that pembrolizumab every 2 weeks is effective and safe for patients with metastatic TNBC after receiving multiple-line therapy. In addition, further trial of KEYNOTE-86^60^^,^^61^ has shown that pembrolizumab monotherapy can prolong OS and/or PFS in metastatic TNBC patients compared with monochemotherapy.

The Impassion130 trial evaluates the therapeutic effect of atezolizumab in combination with nab-paclitaxel and nab-paclitaxel alone as first-line treatment in patients with advanced TNBC. Each group included 451 patients. After a median follow-up of 12.9 months, the atezolizumab group showed longer median PFS (7.2 versus 5.5 months; hazard ratio [HR] = 0.80; 95% CI: 0.69–0.92; p = 0.002). This research revealed that atezolizumab plus nab-paclitaxel prolonged PFS among patients with metastatic TNBC in both the intention-to-treat population and the PD-L1-positive subgroup.

To develop personalized treatments and decrease the demand for chemotherapy or targeted therapy, there is a need to identify novel therapeutic targets or biomarkers for TNBC. circRNAs contribute to the development of various cancer phenotypes. Hence, the next section discusses the potential of the combination of circRNAs and other macromolecules as clinical biomarkers for TNBC.

## Overview of circRNAs

The 5¢ end and 3¢ end of circRNAs are covalently linked, which results in the formation of a closed circular structure. circRNAs are abundantly expressed in the eukaryotic cells.^62^ The role of covalently linked RNA isoforms in cancer initiation and progression was recently reported.^63^ The analysis of circRNAs will improve our understanding of the molecular mechanism underlying tumorigenesis and tumor development. In this review, the biogenesis, characteristics, and functions of circRNAs are described, with a special focus on the regulatory role of circRNA in the initiation and development of TNBC.

### Pathways of circRNA biogenesis

**Figure 1:**
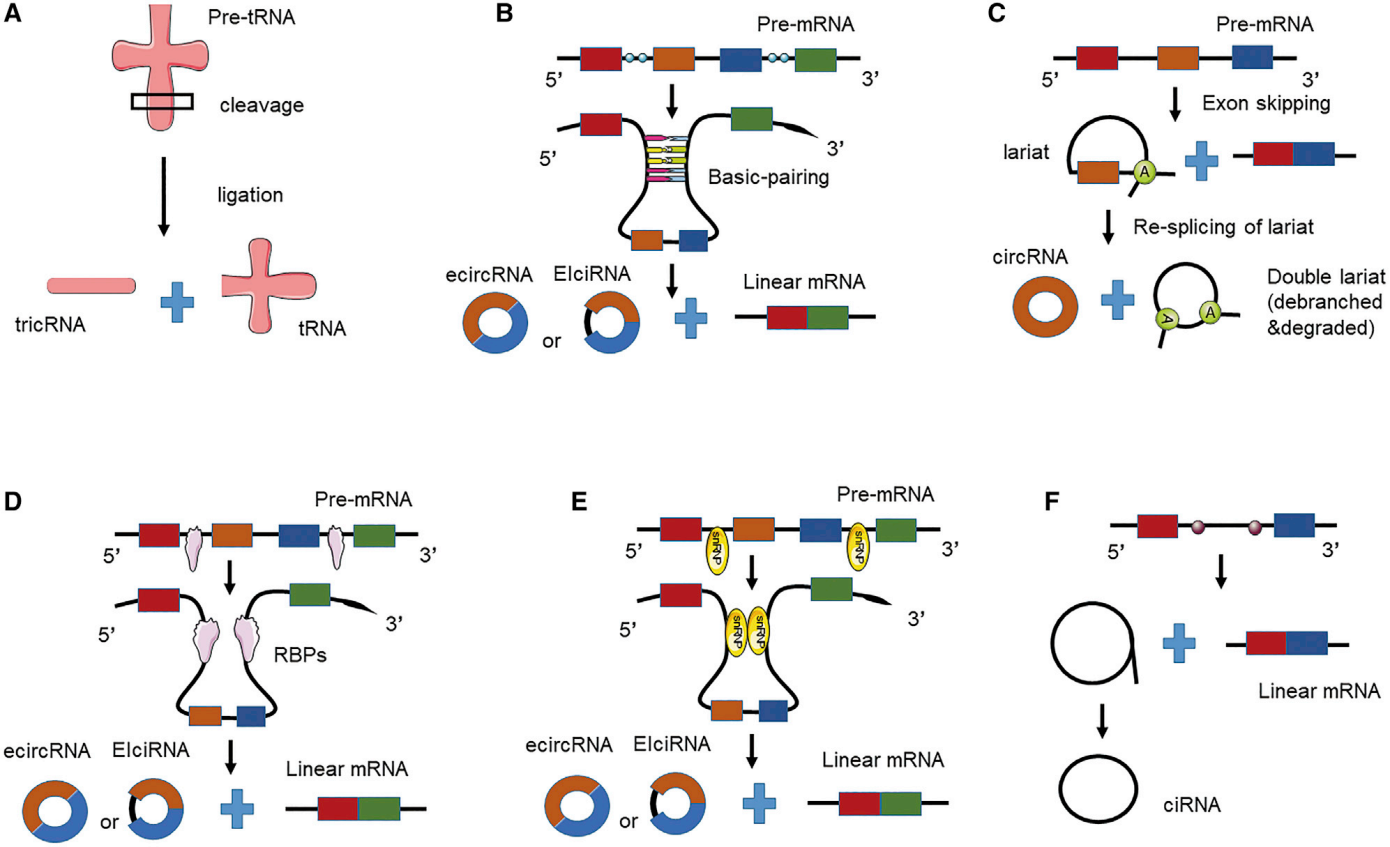
Schematic diagram showing the proposed models of circular RNA (circRNA) biogenesis (A) Transfer RNA splicing-driven circularization. (B) Intron-pairing-driven circularization. Intron pairing brings the corresponding splice signals close to each other to facilitate cyclization. (C) Lariat-driven circularization. Exon skipping results in the production of a mature linear mRNA and an intron lariat containing the middle exon. This lariat is re-spliced to generate a mature circular RNA and a double lariat structure, which subsequently undergoes debranching and degradation. (D) RNA-binding protein (RBP)-driven circularization. RBPs bring the corresponding splice signals close to each other to facilitate cyclization. (E) Small nuclear ribonucleoprotein-dependent circularization. (F) Ribosomal RNA splicing-driven circularization.

Most circRNAs are generated from its precursor mRNA (pre-mRNA), which is catalyzed by RNA polymerase II (Pol II), through a series of processing and splicing events.^64^ Depending on the origin of the donor transcript, several pathways can generate circRNAs (Figure 1). In contrast to linear RNAs, most circRNAs are produced from back-splicing of exons, which results in the formation of a closed circRNA transcript with a 3¢–5¢ phosphodiester bond at the junction site.^27^ Various splicing processes of different donor transcripts generate several types of circRNAs, including exonic circRNAs (ecircRNAs), circular intronic RNAs (ciRNAs), and exon-intron circRNAs (EIciRNAs).^64^ ciRNAs and EIciRNAs are predominantly localized in the nucleus,^65^^,^^66^ while ecircRNAs are localized in the cytoplasm.^67^ Additionally, tRNA intronic circRNAs (tricRNAs) are produced through the splicing of pre-tRNA from the intergenic regions (Figure 1A).^68^

Most studies have focused on the circRNA cyclization process. However, the mechanism underlying circRNA cyclization has not been completely elucidated. The following four models have been proposed for the formation of ecircRNAs or EIciRNAs: intron-pairing-driven circularization (Figure 1B), lariat-driven circularization (Figure 1C), RNA-binding-protein (RBP)-driven circularization^64^ (Figure 1D), and small nuclear ribonucleoprotein (snRNP)-dependent circularization (Figure 1E).

Intron-pairing-driven circularization may be the most frequent mechanism for the formation of ecircRNAs or EIciRNAs.^67^ Reverse complementary sequences, such as ALU repeats,^64^ in the flanking region of exons can pair with each other to form RNA duplexes and consequently form circRNAs through back-splicing. The upstream 3¢ splice donor (splice acceptor) site is covalently joined with a downstream 5¢ splice site (splice donor).^69^^,^^70^ Alternatively, the retention of the introns flanking the exon results in the formation of EIciRNAs, whereas the removal of these introns results in the formation of ecircRNAs. In some cases, the stabilized RNA duplex may inhibit the intron-pairing-driven circularization through an unknown mechanism.^71^

Recent global transcriptome analyses^72^ revealed that the lariat-driven circularization involved the formation of folding regions, which involved exon-skipping events. Hence, a linear mRNA and a circRNA can be generated from a single pre-mRNA. Barrett et al.^73^ demonstrated the coupling between circRNA biogenesis and exon skipping. At the *Saccharomyces pombe* mrps16 gene, splicing of exon 1 to exon 3 releases an intron lariat containing exon 2, which is subsequently re-spliced to generate a mature circRNA and a double lariat structure that undergoes debranching and degradation. The second splicing of mrps16 intron lariat (but not of other lariats) may be dependent on the speed of lariat debranching and degradation or topological effects. Thus, lariat-driven circularization provides a model for producing circRNAs in the absence of intronic repeats.

Additionally, the sequences in the intron flanking of exons can bind to RBPs (as *trans*-acting activators) and form a bridge to stabilize the head-to-tail connection between the splice donor and acceptor sites, which leads to the formation of circRNAs.^74^ Additionally, circRNAs can be synthesized through alternative splicing events by a sequential assembly of snRNPs.^74^

To generate intronic circRNAs (Figure 1F), specific introns containing reverse complementary sequences can pair to form a lariat, whereas other lariats immediately undergo debranching. The lariats formed from the introns containing reverse complementary sequences subsequently undergo 3¢ end tail degradation to form ciRNAs. These differing models of ciRNA and ecircRNA biogenesis indicate the importance of the structure of circRNAs. CiRNAs are characterized by the presence of a 2¢–5¢ junction, which arises from a residue of the original lariat structure. In contrast, ecircRNAs have a 3¢–5¢ linkage at the splice branch point.

### Turnover of circRNAs

Compared to linear counterparts, circRNAs are speculated to be highly stable due to their covalently closed structures being resistant to degradation by exoribonuclease, thus making them ideal candidates for biomarker development.^26^ Although back-splicing is inefficient, some circRNAs can be accumulated to high levels post-transcriptionally. A study has shown that the median half-life of 60 circRNAs in mammary cells was 18.8–23.7 h, compared with 4.0–7.4 h for their corresponding linear transcripts.^75^ The steady-state levels of circRNAs were observed to be positively correlated with their nascent levels in cells with similar mitotic cycles, indicating that the detection of steady-state circRNAs in a cell- and/or tissue-specific manner likely reflects the endogenous synthesis of circRNAs: the more nascent circRNAs are produced, the higher the steady-state levels of circRNAs detected.

To evaluate the stability of circRNAs, Zheng et al.^76^ quantified the ratio of circular-to-linear SEPT9 gene by quantitative real-time polymerase chain reaction (quantitative real-time PCR). circSEPT9 showed resistance to exoribonuclease and was thus significantly more stable than its linear counterparts. In an orthogonal method to assess the stability of circSEPT9, they compared concentrations of circular and linear transcripts in TNBC tumor cells that were treated with actinomycin D, a transcription inhibitor, over time. In these tumor cells harvested after actinomycin D treatment, circRNA levels increased while mRNA levels decreased, thus demonstrating the relatively higher stability of circular transcripts.

## circRNAs and TNBC

### Molecular mechanisms of circRNAs in TNBC

**Figure 2:**
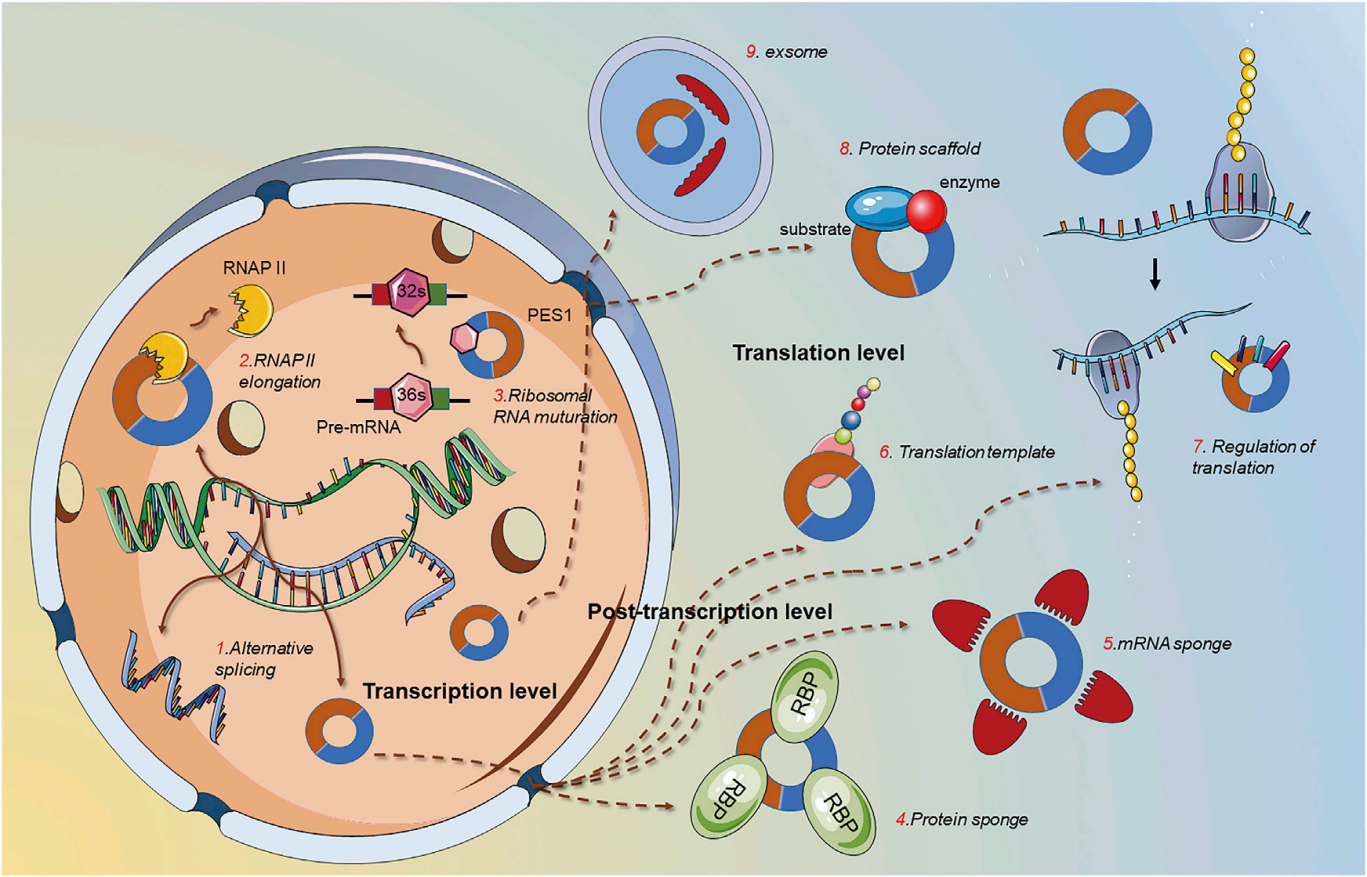
Schematic diagram of circRNA functions in eukaryotes circRNAs function as microRNA or RNA-binding protein sponges to regulate the expression of relevant elements. circRNAs act as protein scaffolds to promote the binding of a substrate to an enzyme. Additionally, circRNAs can modulate gene expression at the transcription and translation levels. Listed are some gene expression regulatory functions of circRNAs.

Various studies^77^ have reported that circRNAs have potential therapeutic and functional roles at the molecular level. circRNAs can function as miRNA sponges, regulate transcription and alternative splicing of the gene from which they are derived, interact with proteins, and serve as templates for translation. Further details on the function of circRNAs are described in Figure 2.

#### miRNA sponge

The competing endogenous RNA (ceRNA) hypothesis states that RNAs regulate miRNA activities via sequestration, leading to mediation of the post-transcriptional dysregulation of miRNA target mRNAs indirectly.^78^ Prominent attention has been paid to this hypothesis as a unifying function of lncRNAs, pseudogene transcripts, and circRNAs, as well as an alternative function of mRNAs.^79^ There are over 500 miRNA-encoding genes in the human genome, and miRNAs derived from individual gene families are capable of targeting or affecting the stability and translation of multiple diverse mRNAs.^80^^,^^81^ circRNAs can sponge miRNAs through multiple binding sites for cognate target miRNA, availing them to modulate miRNA activity and upregulate the expression of miRNA target genes.

The positive or negative roles of circRNAs identified to date in TNBC are primarily based on their miRNA-sponging activity. Some circRNAs even bind several miRNAs, as exemplified by circRAD18, which contains target sites for miR-208a and miR-3164.^82^ As shown in a study by Zou et al.,^82^ circRAD18 upregulates IGF1 and FGF2 expression to promote TNBC progression. Knockdown of circRAD18 stimulated cell apoptosis, significantly inhibited tumor growth, and suppressed cell proliferation and migration by targeting miR-208a and miR-3164 in functional and xenograft experiments through luciferase reporter assays.^82^

Additionally, specific circRNAs increase the transcription of target genes^83^ or protect their homologous mRNAs from miRNA-mediated degradation by inhibiting miRNA activity.^84^ For instance, He et al.^85^ revealed that circGFRA1, a ceRNA that binds miR-34a, regulates the expression of target gene GFRA1 to promote TNBC proliferation and invasion. The rescue experiment results showed that the small hairpin RNA-induced circGFRA1 knockdown in TNBC cells dramatically inhibited the resistance of TNBC cells to paclitaxel (PTX) due to the inhibition of miR-361-5p.^86^

In addition, few circRNAs may indirectly activate or inactivate pivotal signaling pathways, such as the Wnt/β-catenin pathway,^87^ Stat3 pathway,^76^ TLR pathway,^86^ etc., by inhibiting miRNAs. circITCH functions as a sponge for miR-214 and miR-17 to increase expression of its ITCH linear isoform and inactivate Wnt/β-catenin signaling.^87^ These findings indicated that miRNA sponging is the major function of circRNAs in malignant tumors or that this activity is the most widely studied. However, the importance of this mechanism is unclear, as not all circRNAs contain the binding sites for miRNAs.

#### Regulation of gene transcription

Some studies have focused on the regulation of gene expression by circRNAs at the transcriptional level. circRNAs, which are mainly localized to the nucleus, can regulate gene expression by interacting with the binding site.^27^ Li et al.^65^ used a genomic approach to identify circEIF3J and circPAIP2 in the nucleus. These ElciRNAs upregulated the expression of their parental genes in *cis*. This indicated the presence of a transcriptional regulatory mechanism, which involves specific RNA-RNA interaction between U1 small nuclear RNA (snRNA) and ElciRNAs.^65^ Furthermore, circMBL can integrate with its parental protein to inhibit further production of the MBL transcript in a regulatory feedback loop.^88^ Additionally, a similar study demonstrated that circANKRD52 and circSIRT7 can regulate transcription in *cis* and consequently upregulate the transcription of their parental gene by interacting with RNA Pol II complexes.^66^ However, the specific mechanism underlying these circRNA functions on upstream regulators is still unknown.

In addition, some circRNAs can induce the proliferation and progression of TNBC by regulating the transcription of genes involved in cancer-associated signaling pathways. Recent studies have demonstrated that circRNA_069718 regulated the genes related to the Wnt/β-catenin pathway (β-catenin, c-*myc*, and cyclin D1) at both mRNA and protein levels in TNBC cells, which is a critical cascade associated with cancer progression and capable of inducing epithelial-mesenchymal transition (EMT).^89^ Interestingly, circRNA_069718 inhibition decreased TNBC cell proliferation and invasion *in vitro*. These results demonstrated that therapeutic targeting of circRNA_069718 may inhibit TNBC tumorigenesis and metastasis.

#### Interaction of protein

Various studies have demonstrated that some circRNAs can function as protein chaperones to regulate gene expression by interacting with related proteins. For example, circANRIL can bind directly to pescadillo homolog 1 in 60S-preribosomal assembly and exert atheroprotective effects by regulating the maturation of ribosomal RNA (rRNA) and controlling the progress of atherogenesis.^90^ Similarly, low levels of circFoxo3 are associated with RBPs and prevent their nuclear translocation.^91^ circFoxo3 could form a ternary complex by binding to FAK and HIF-1α proteins and consequently inhibit their nuclear translocation, which suppresses cell cycle progression.^91^

#### Translation template

Due to the deficiency of essential elements for cap-dependent translation, most circRNAs are regarded as being noncoding. But, in fact, circRNAs equipped with initial codon sites (AUG), an open reading frame (ORF), and internal ribosome entry site (IRES) elements may work as translation templates to fabricate particular peptides with specific functions.^67^^,^^92^^,^^93^ For example, Ye et al.^94^ identified that overexpression of circFBXW7 obviously suppressed cell proliferation, migration, and tumor growth for TNBC cells in both *in vitro* and *in vivo* assays. The molecular mechanism of circFBXW7 not only functions as a sponge of miR-197-3p to suppresses TNBC growth but also encodes a novel 21-kDa protein, termed FBXW7-185aa, to inhibit the progress of tumor cells.^94^ As circFBXW7-derived peptides (FBXW7-185aa) are usually shorter than their congenetic linear mRNA-translated proteins and lacking elementary functional domains, they might serve as dominant-deleted protein variants, decoys, or regulators of alternative proteins.^95^ Importantly, the peptide FBXW7-185aa inhibited the proliferation and migration abilities and regulated the cell cycle of TNBC cells by increasing the abundance of FBXW7 and inducing c-Myc degradation.^94^^,^^96^ Furthermore, N6-methyladenosine enriched in the circRNA sequences can initiate translation by recruiting eIF4G2 and YTHDF3.^97^

### Biological functions of circRNAs in TNBC

**Figure 3:**
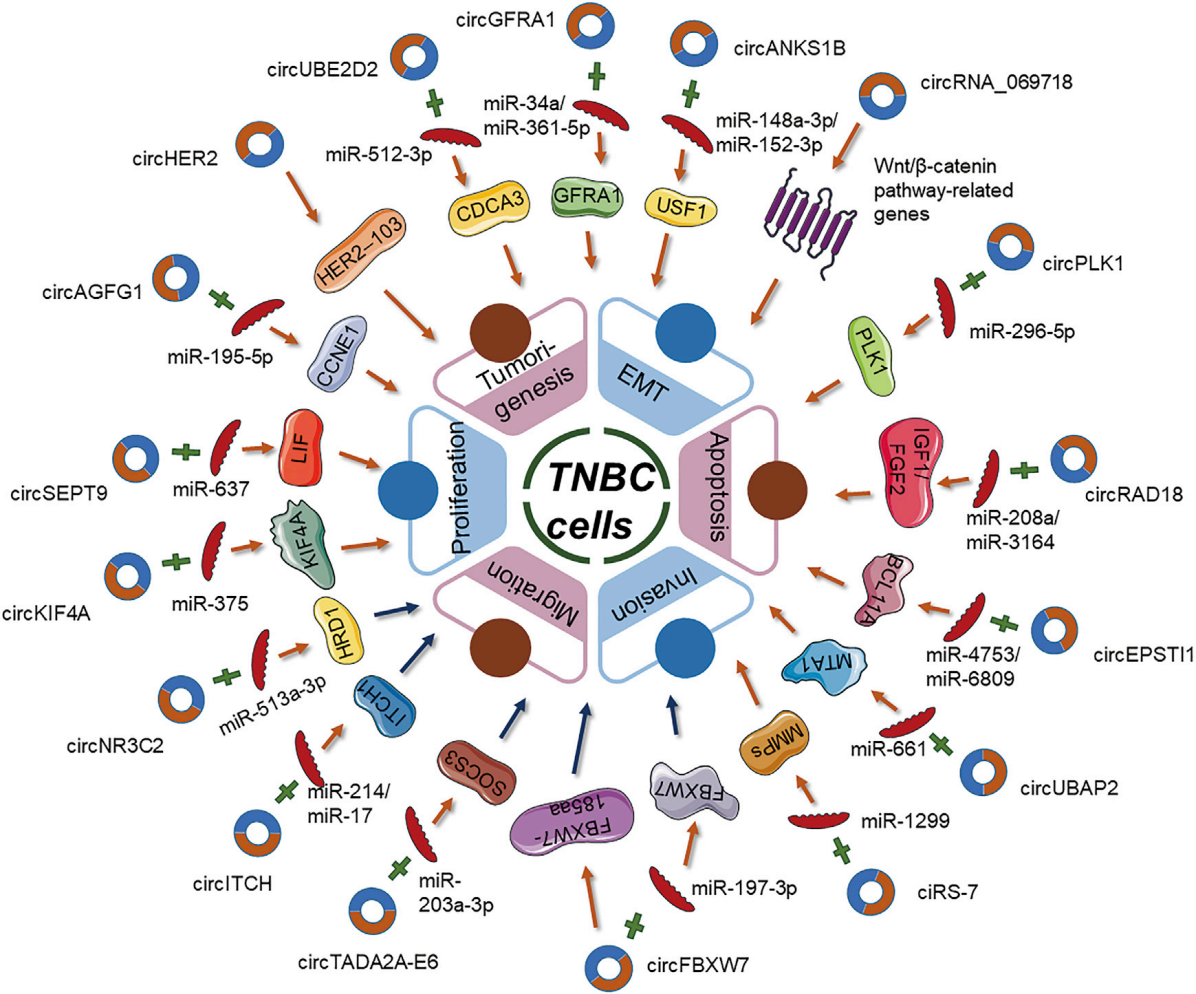
Schematic diagram of the roles and regulatory pathways of triple-negative breast cancer (TNBC)-associated circular RNAs TNBC-related circRNAs have been implicated in several hallmarks of cancer. The involvement of these circRNAs in the miRNA-associated gene regulatory pathways has been displayed.

Recently, the role of circRNAs in the regulation of genes involved in clinical cancers has been examined. However, the profiles of circRNAs in TNBC could be concerned depending on the invasiveness of these subgroup tumors. Several bioinformatic tools have been developed to accelerate the investigations into the functions and the underlying mechanisms of circRNAs. Novel aberrant circRNAs have been validated as key molecular regulators in TNBC (Table 2). These circRNAs have major roles in the carcinogenesis of TNBC, including the regulation of tumor proliferation, apoptosis, invasion, migration, chemoradiation resistance, or activation of EMT, based on the results of *in vitro* and *in vivo* functional experiments. This section focuses on the correlation between these circRNAs and their biological functions in TNBC. Most studies examining the function of circRNAs in cancer have focused on the ceRNA hypothesis.^78^ circRNAs positively or negatively regulate the proliferation, invasion, and tumorigenesis of TNBC through the circRNA-miRNA-mRNA axis (Figure 3).

**Table 2:** Main circRNAs associated with TNBC

circRNA	Study (year) reference	Expression change	Hallmarks of cancer	Target microRNA	Target gene	Mechanism
circSEPT9	Zheng et al. (2020)^76^	↑	proliferation (+); invasion (+); migration (+); apoptosis (−)	miR-637	LIF	circSEPT9 functions as a **sponge** of miR-637 to downregulate the LIF and activate LIF/Stat3 signaling pathway to promote TNBC progression
circUBE2D2	Dou et al. (2020)^84^	↑	tumorigenesis (+); proliferation (+); invasion (+); migration (+)	miR-512-3p	CDCA3	circUBE2D2 functions as a **sponge** of miR-512-3p for upregulation of CDCA3 to promote TNBC progression and doxorubicin resistance
circHER2	Li et al. (2020)^98^	↑	tumorigenesis (+); proliferation (+); invasion (+)	NA	HER2-103	CircHER2 **encodes** a HER2-103 **protein** to promote TNBC progression
circAGFG1	Yang et al. (2019)^99^	↑	tumorigenesis (+); proliferation (+); invasion (+); migration (+)	miR-195-5p	CCNE1	circAGFG1 acts as a **ceRNA** of miR-195-5p to relieve the repressive effect of miR-195-5p toward CCNE1
circKIF4A	Tang et al. (2019)^100^	↑	proliferation (+); migration (+)	miR-375	KIF4A	circKIF4A functions as a **sponge** of miR-375 to regulate the expression of KIF4A
circPLK1	Kong et al. (2019)^101^	↑	proliferation (+); migration (+)	miR-296-5p	PLK1	circPLK1 functions as a **sponge** of miR-296-5p to regulate PLK1
circRNA_069718	Zhang et al. (2019)^89^	↑	proliferation (+); invasion (+); EMT (+)	NA	Wnt/β-catenin pathway-related genes	circRNA_069718 **regulates** the expression levels of Wnt/β-catenin pathway-related **genes** (e.g., β-catenin, c-myc, and cyclin D1)
circRAD18	Zou et al. (2019)^82^	↑	proliferation (+); migration (+); apoptosis (−)	miR-208a/miR-3164	IGF1/FGF2	circRAD18 functions as a **sponge** of miR-208a and miR-3164 to upregulate IGF1 and FGF2 expression and promote TNBC progression
circANKS1B	Zeng et al. (2018)^83^	↑	invasion (+); migration (+); EMT (+)	miR-148a-3p/miR-152-3p	USF1	circANKS1B functions as a **sponge** of miR-148a-3p and miR-152-3p to increase the expression of transcription factor USF1 and promote EMT
circEPSTI1	Chen et al. (2018)^143^	↑	proliferation (+); invasion (+); apoptosis (−)	miR-4753/miR-6809	BCL11A	circEPSTI1 functions as a **sponge** of miR-4753 and miR-6809 to regulate BCL11A expression and affect TNBC proliferation and apoptosis
circUBAP2	Wang et al. (2018)^144^	↑	proliferation (+); migration (+); apoptosis (−)	miR-661	MTA1	circUBAP2 functions as a **sponge** of miRNA-661 to increase the expression of the oncogene MTA1
ciRS-7	Sang et al. (2018)^102^	↑	invasion (+); migration (+)	miR-1299	MMPs	ciRS-7 functions as a **sponge** of miR-1299 to enhance the expression of MMPs
circGFRA1	He et al. (2017)^85^/Zheng et al. (2021)^86^	↑	proliferation (+); invasion (+); apoptosis (−)	miR-34a/miR-361-5p	GFRA1	circGFRA1 functions as a **sponge** of miR-34a to regulate GFRA1 expression/functions as a sponge of miR-361-5p for promoting the resistance of TNBC to PTX
circNR3C2	Fan et al. (2021)^145^	↓	proliferation (−); invasion (−); migration (−); EMT (−)	miR-513a-3p	HRD1	circNR3C2 functions as a **sponge** for miR-513a-3p to downregulate HRD1 to promote tumor growth and metastasis
circITCH	Wang et al. (2019)^146^	↓	proliferation (−); invasion (−); migration (−)	miR-214/miR-17	ITCH1	circITCH functions as a **sponge** for miR-214 and miR-17 to increase expression of its ITCH linear isoform and inactivate Wnt/β-catenin signaling
circTADA2A-E6	Xu et al. (2019)^103^	↓	proliferation (−); invasion (−); migration (−);	miR-203a-3p	SOCS3	circTADA2A-E6 functions as a **sponge** of miR-203a-3p to restore the expression of SOCS3, resulting in a less aggressive oncogenic phenotype
circFBXW7	Ye et al. (2019)^94^	↓	proliferation (−); invasion (−); migration (−);	miR-197-3p	FBXW7	circFBXW7 functions as a **sponge** of miR-197-3p to suppresses TNBC growth **or encodes** the FBXW7-185aa **protein** to inhibit the proliferation and migration abilities of TNBC cells

↑, upregulated; ↓, downregulated; (+), promoted; (−), inhibited; EMT, epithelial-mesenchymal transition; LIF, leukemia inhibitory factor; NA, not applicable.

#### Oncogene role of circRNAs in TNBC

As a rule, circRNAs upregulated in solid tumor are regarded as oncogenes to advocate tumorigenesis, cell proliferation, migration, invasion, and metastasis, yet suppressing cell cycle arrest and apoptosis. For example, the expression of circUBE2D2 in TNBC was upregulated when compared with that in the mammary epithelial cell line MCF-10A.^84^ The knockdown of circUBE2D2 repressed proliferation, migration, and invasion of TNBC cells *in vitro* and impaired tumorigenesis of TNBC *in vivo*. Mechanistically, circUBE2D2 sponges miR-512-3p, which results in the upregulation of CDCA3 expression. Additionally, rescue experiments have revealed that downregulated miR-512-3p expression may markedly mitigate the circUBE2D2 depletion-mediated tumor-suppressive effects. Furthermore, this study revealed the role of the circUBE2D2/miR-512-3p/CDCA3 axis in conferring doxorubicin resistance to TNBC. EMT is critical for the invasion and metastasis of multiple cancers, including TNBC, as it involves the downregulation of biomarkers and impairs polarity and adhesion capacity of epithelial cells.^104^ TGF-β signaling also played a pivotal role in inducing EMT, which is crucial for BC progression and heterogeneity.^105^ A recent study demonstrated the role of a novel circRNA, circANKS1B, which is upregulated in TNBC. circANKS1B promotes the expression of the transcription factor USF1 and upregulates the transcription of TGF-β1. Consequently, circANKS1B induces EMT by sponging miR-148a-3p and miR-152-3p.^83^ Moreover, upregulated circANKS1B expression, which promotes migration and invasion of cancer cells, served as an independent risk factor for poor OS in patients with BC.^83^ Other circRNAs, such as circAGFG1, circKIF4A, and circPLK1, are also reported to promote tumorigenesis in TNBC by sponging miRNAs.^99^, ^100^, ^101^

Other mechanisms of circRNAs also exert therapeutic effects in patients with TNBC. For example, circHER2, which encodes a novel protein called HER2-103, promotes downstream malignant phenotypes by interacting and activating EGFR/HER3 and promoting AKT phosphorylation.^98^ Further studies are needed to identify the standard regimen, as this mode of circHER2/HER2-103 regulation will contribute to the chemoresistance to pertuzumab, a known target antibody of HER2.^98^

#### Tumor suppressor activity of circRNAs in TNBC

Generally speaking, circRNAs downregulated in TNBC typically have effects on tumor-suppressor activities inhibiting cell proliferation, migration, and invasion but promoting cell cycle arrest and apoptosis. Recently, circITCH was reported to function as a novel tumor suppressor in TNBC.^87^ circITCH sponges miR-214 and miR-17 and consequently upregulates the expression of its ITCH linear isoform, which downregulates the Wnt/β-catenin signaling pathway. The overexpression of circITCH markedly suppressed the proliferation, invasion, and metastasis of TNBC both *in vitro* and *in vivo*.^87^ Fan et al.^106^ confirmed that circNR3C2 is remarkably downregulated in TNBC, and its expression is negatively correlated with the distant metastasis and lethality of invasive breast carcinoma. *In vitro* and *in vivo*, the overexpression of circNR3C2 leads to an overwhelming escalation of the tumor-suppressive effects of gene HRD1 via sponging miR-513a-3p.^106^ HRD1 is significantly underexpressed in TNBC against other subtypes and is identified to bind vimentin with high probability. Vimentin, a type 3 intermediate filament protein, plays a pivotal role in maintaining the cell shape and stabilizing cytoskeletal interactions.^107^ Vimentin is a vital mesenchymal marker in the very center of the EMT process and is regarded as an organizer of significant proteins involved in cell attachment, through which increased cellular invasion and migration lead to the metastasis of BC.^108^ circNR3C2 subsequently facilitated the inhibition of cell proliferation, migration, invasion, and the EMT process in TNBC cells via inducing polyubiquitination-mediated proteasomal degradation of vimentin.^106^ Xu et al.^103^ used microarrays to identify 235 differentially expressed circRNAs in BC. The authors focused on circTADA2A-E6, which was downregulated in TNBC and was associated with survival rate. Bioinformatic analysis revealed that the overexpression of circTADA2A-E6 promotes the expression of its target gene SOCS3 by sponging miR-203a-3p and consequently decreases the malignant phenotype. Although rare circRNAs have been reported to play a positive and protective role in TNBC-related signaling pathways, further studies are needed to examine the role of circRNAs in the pathogenesis of TNBC.

#### Modulating chemoresistance of circRNAs in TNBC

Chemoresistance or radiation resistance is a major limitation for determining the optimal clinical treatment. However, the role of circRNAs in determining the sensitivity of tumors to chemotherapy has piqued the interest of the scientific community. Doxorubicin-based chemotherapy is the most common treatment regimen for TNBC.^109^ However, drug resistance develops after periodic application. A recent study^84^ demonstrated that the overexpression of circUBE2D2 decreased cell viability, while the silencing of circUBE2D2 enhanced doxorubicin-induced apoptosis. The authors further demonstrated that the knockdown of circUBE2D2 can confer doxorubicin resistance by modulating the miR-512-3p/CDCA3 axis. Gao et al.^110^ revealed that the expression of hsa_circ_00006528 in adriamycin (ADM)-resistant breast cancer (BC) tissues was higher than that in ADM-sensitive tissues, which indicated that it is a promising candidate for preventing chemoresistance in BC. Although circRNAs can contribute to improving the clinical management of chemotherapy resistance, there are limited studies on their functions. Further studies are needed to elucidate the mechanisms underlying the therapeutic potential of circRNAs.

## Clinical potential of circRNAs in TNBC

Recently, much attention has been paid to early detection and quantification of circRNAs as biomarkers in tumor biopsies. Compared with normal controls, spatiotemporal differences of specific circRNAs can be found in the mammary tissue and body liquid from patients with TNBC. Thus, they are ideal candidates as biopsy biomarkers for the diagnosis of TNBC in tumor biopsies. Additionally, circRNAs related to OS and clinicopathological characteristics may serve as an independent prognostic factor for patients with TNBC.^26^

### circRNAs as potential diagnostic and prognostic biomarkers for TNBC

Early screening and diagnosis of TNBC can improve the efficacy of cancer therapy and decrease the mortality of patients with TNBC. Hence, there is a need to identify biomarkers with high specificity, sensitivity, and predictive power for the early diagnosis of TNBC. An optimal biomarker is defined by the National Cancer Institute as “a biological molecule found in the blood, other body fluids, or tissues that are a sign of a normal or abnormal process, or of a condition or disease.”^27^ Recent studies have examined the correlation between aberrant expression of specific circRNAs, disease specificity, and clinical relevance in TNBC (Table 3). Compared to miRNAs and lncRNAs, circRNAs can be potential liquid biopsy biomarkers for noninvasive procedures. This is because of the following unique characteristics of circRNAs: long half-life (>48 h); high stability; resistance to RNase R digestion due to the circular structure with no 5¢ end cap and 3¢ poly (A) tails;^111^ expression in tissue- and developmental-stage-specific manners;^112^ abundant expression in various body fluids, such as the blood, plasma, serum, or exosomes;^26^^,^^27^ and detection by cost-effective methods, such as quantitative real-time PCR.^26^ Thus, distinct expression patterns of circRNAs present as predictive biomarkers when the medical technology allows. For example, Yang et al.^99^ demonstrated that circAGFG1 can discriminate patients with TNBC from healthy controls. In accordance with the RNA sequencing (RNA-seq) data by quantitative real-time PCR, the results revealed that circAGFG1 was identified as being broadly increased in TNBC tissues in comparison to adjacent non-tumor tissues and normal cell line. Receiver operating characteristic (ROC) curve analysis proved that circAGFG1 could sensitively discriminate TNBC from non-cancerous tissues, indicating its significant value in diagnosing TNBC.^99^ Inversely, compared to healthy control groups, Xu et al.^103^ documented the distinct downregulated levels of circTADA2A-E6 in patients with TNBC. Interestingly, the area under the curve (AUC) was 0.8554 for circTADA2A-E6, indicating that 85.5% of patients with TNBC had lower expression levels of circTADA2A-E6 than healthy individuals. The AUC of circTADA2A-E6 suggested that it may be a potential noninvasive biomarker for the detection of TNBC.^103^ Importantly, the utilization of the combination of circRNA and its target mRNA or downstream proteins can aid in increasing the diagnostic accuracy.

**Table 3:** Summary of associations between circRNAs and diagnosis and prognosis in TNBC

circRNA	Study (year) reference	Length	Source	Clinical stage relatedness	Prognosis
circSEPT9	Zheng et al. (2020)^76^	645 bp	4 pairs of tumor and adjacent noncancerous tissues	circSEPT9 positively correlated with TNM stage, metastasis, and chemoresistance	high circSEPT9 predicted a poor prognosis
circUBE2D2	Dou et al. (2020)^84^	280 bp	66 pairs of tumor tissues	circUBE2D2 positively correlated with TNM stage, lymph node metastasis, and chemoresistance	high circUBE2D2 predicted a poor prognosis
circHER2	Li et al. (2020)^98^	676 bp	59 patients	circHER2 positively correlated with tumor grade and metastasis	high circHER2 predicted a poor prognosis
circAGFG1	Yang et al. (2019)^99^	527 bp	4 pairs of tumor tissues	circAGFG1 positively correlated with tumor grade, pathological grade, and lymph node invasion	high circAGFG1 predicted poor OS
circKIF4A	Tang et al. (2019)^100^	355 bp	240 tumor tissues	circKIF4A positively correlated with tumor grade, lymph node invasion, and metastasis	high circKIF4A predicted poor OS and DFS
circPLK1	Kong et al. (2019)^101^	1,708 bp	240 tumor tissues	circPLK1 positively correlated with tumor grade, invasion, lymph node stage, and metastasis	high circPLK1 predicted poor OS and DFS
circRNA_069718	Zhang et al. (2019)^89^	590 bp	35 pairs of tumor tissues	circRNA_069718 positively correlated with lymph node stage and metastasis	high circRNA_069718 predicted poor OS
circRAD18	Zou et al. (2019)^82^	756 bp	126 tumor tissues	circRAD18 positively correlated with T stage, clinical stage, and pathological grade	high circRAD18 predicted poor OS
circANKS1B	Zeng et al. (2018)^83^	459 bp	165 tumor tissues	circANKS1B positively correlated with lymph node stage and metastasis	high circANKS1B predicted poor OS
circEPSTI1	Chen et al. (2018)^143^	375 bp	240 tumor tissues	circEPSTI1 positively correlated with tumor grade, invasion, lymph node stage, and metastasis	high circEPSTI1 predicted poor OS and DFS
circUBAP2	Wang et al. (2018)^144^	747 bp	78 tumor tissues	circUBAP2 positively correlated with tumor size, TNM grade, lymph node invasion, and metastasis	high circUBAP2 predicted poor OS
circGFRA1	He et al. (2017)^85^/Zheng et al. (2021)^86^	427 bp	222 tumor tissues	circGFRA1 positively correlated with tumor grade, lymph node invasion, and metastasis	high circGFRA1 predicted poor OS and DFS
circNR3C2	Fan et al. (2021)^145^	1,759 bp	70 tumor tissues	circNR3C2 negatively correlated with tumor growth and metastasis	low circNR3C2 predicted poor prognosis
circITCH	Wang et al. (2019)^146^	NA	91 tumor tissues	circITCH negatively correlated with tumor grade, lymph node invasion, and metastasis	low circITCH predicted poor OS
circTADA2A-E6	Xu et al. (2019)^103^	158 bp	121 tumor tissues	circTADA2A-E6 negatively correlated with lymph node invasion and metastasis	low circTADA2A-E6 predicted poor DFS
circFBXW7	Ye et al. (2019)^94^	1,227 bp	473 tumor tissues	circFBXW7 negatively correlated with tumor grade and lymph node invasion	low circFBXW7 predicted poor OS and DFS

OS, overall survival; DFS, disease-free survival; LIF, leukemia inhibitory factor; NA, not applicable.

Additionally, specific circRNAs listed in Table 3 could represent attractive prognostic biomarkers. Patients with dysregulated levels of circRNAs had conspicuously different prognoses, as evidenced by clinicopathological characteristics (e.g., TNM stage, lymph node metastasis, distant metastasis, and postoperative recurrence) and DFS or OS. For instance, the expression of circKIF4A was upregulated, which was positively associated with poor survival, in TNBC by quantitative real-time PCR analyses.^100^ The results of the luciferase reporter and RNA immunoprecipitation assays revealed that circKIF4A promoted tumor proliferation and migration through the regulation of KIF4A expression by sponging miR-375. This indicated that circKIF4A is associated with tumor grade, lymph node invasion, and distal metastasis, indicating it is an independent prognostic indicator for TNBC. Similarly, Kong et al.^101^ demonstrated that upregulated circPLK1 expression was significantly and positively correlated with large tumor size, invasion, lymph node stage, and metastasis. This indicated that circPLK1 has critical roles in TNBC progression. Furthermore, Kaplan-Meier analysis of OS and DFS curves revealed that the upregulated levels of circPLK1 may lead to poor survival in patients with TNBC. In contrast, circFBXW7 expression, which was negatively correlated with tumor stage and lymph node metastasis, can be an independent prognostic factor for patients with TNBC.^94^ The upregulated expression of circFBXW7 markedly suppressed cell proliferation, migration, and tumor progression *in vitro* and was validated *in vivo* based on the decreased OS and DFS.

Although circRNAs are stable in the peripheral blood, some studies have suggested that circRNAs in the peripheral blood can be used as biomarkers to distinguish healthy individuals from patients with cancer.^113^^,^^114^ However, there are several limitations associated with the clinical application of circRNAs as biomarkers. Multi-center prospective studies are required before clinical application of circRNAs as biomarkers. Additionally, the techniques used to detect circRNAs must be scaled up to an industrial scale. Furthermore, the collection timing and cutoff values for circRNA must be standardized. These findings indicate the potential of circRNAs as diagnostic and prognostic biomarkers for TNBC.

### circRNAs as potential therapeutic targets for TNBC

According to our review, dysregulated circRNAs are zealously involved in carcinogenesis and progression of TNBC, and the silence of these circRNAs proved to be counter-productive *in vitro* and *in vivo*. Thus, precise interfering RNAs designed precisely to target the unique back-spliced junction (BSJ) of upregulated circRNAs would exert an influence on anti-tumor treatment. Conversely, increasing the concentration of downregulated circRNAs in TNBC would also yield substantial anti-cancer effects. We benefit from regarding functional circRNAs on aspects of pharmacokinetic properties partially because of their high stability and long half-life;^115^ however, there is still a long way to go before putting the circRNAs into clinical use as a pharmaceutic preparation.

By reviewing the accumulated research listed in Tables 2 and 3, we concluded that tumor growth and metastasis was inhibited efficaciously by small interfering RNAs (siRNAs) or short hairpin RNAs (shRNAs) specifically targeting oncogenic circRNAs in patient-derived xenograft (PDX) mouse models. For instance, Sang et al.^102^ demonstrated that tail vein injection of diverse siRNAs specifically targeting ciRS-7 suppresses the migration and invasion of TNBC cells in TNBC PDX-mouse models, manifesting that the oncogenic ciRS-7 may serve as a potential therapeutic target for TNBC treatment. They also reported that the knockdown of ciRS-7 suppressed the liver and lung metastases of TNBC cells *in vivo* by functioning as a ceRNA to sponge miR-1299. Thus, ciRS-7 exerted tumor suppressor effects by regulating the members of matrix metalloproteinases. Hence, targeting the ciRS-7/miR-1299/MMP axis can be a novel therapeutic strategy for TNBC. These findings further indicate that RNA circularization is a potential therapeutic strategy for TNBC. Furthermore, as circRNAs can sponge miRNAs and RBPs, they can function as potential therapeutic vectors. Various methods have been proposed to inhibit or restore circRNA sponging function,^30^ including inhibition of siRNAs, anti-sense oligonucleotides, CRISPR-Cas9-mediated knockout, molecular interaction and saturation of the conserved binding sites, and reintroduction of circRNAs to restore dysregulated proliferation or induce apoptosis of tumor cells.

When it comes to the reintroduction or delivery of therapeutic circRNAs, their unique cellular stability enables their application as a promising target vector. Specifically designed artificial circRNAs can sequester oncogenic miRNAs and RBPs to inhibit tumor progression. Extracellular vesicles and nanoparticles can be used to enhance the delivery of circRNAs.^116^ Generally, circRNAs have potential applications in gene therapy in the future.

Detecting the expression of circRNAs related to chemoresistance may be important for predicting the sensitivity of patients with TNBC to chemotherapy in the clinic. Moreover, interference with such circRNAs might also promote the sensitivity of patients with TNBC to chemotherapy. Zheng et al.^86^ verified that circGFRA1 functions as a sponge of miR-361-5p for promoting the resistance of TNBC to PTX through the TLR4 pathway. *In vitro*, circGFRA1 silenced upregulated the expression of miR-361-5p in TNBC PDX-mouse models treated with PTX. Western blot results showed a decrease in TLR4 expression in PTX-treated mice after lentivirus (LV-si-circGFRA1) injection. Also, the size of the tumor was reduced. Conversely, cicGFRA1 and TLR4 were highly expressed in PDX-mice treated with PTX only, and the size of tumor was increased.

Finally, the recent usage approval of ICIs in TNBC by the FDA has reinvigorated the enthusiasm of researchers to understand the connection between immunotherapy and therapeutic circRNAs.^21^ PD-1/PD-L1 is an important component of tumor immunosuppression, while immunosuppression is a type of mechanism for tumor immune escape.^117^ The interaction between PD-1 and PD-L1 can suppress effector T lymphocytes effectively, triggering the tumor immune escape.^118^ As the high PD-L1 expression and amplification of CD274 were found in most TNBC,^54^ developing drugs that block the PD-L1 pathway is an attractive potential cancer immunotherapy. Recent studies demonstrated that circRNAs were able to regulate the expression of PD-L1 via serving as ceRNAs, promoting the tumor escape immune surveillance.^119^ However, current clinical trials about therapeutic circRNAs targeting PD-L1 are based on CRC^120^ and non-small cell lung cancer (NSCLC),^121^ rarely involving TNBC. For example, Tanaka et al.^122^ proved that interventional methods targeting CDR1-AS would increase the PD-L1 levels in CRC cells, inducing T cell apoptosis and inhibiting T cell activation and proliferation, eventually resulting in cancer immune escape. In addition, accumulating evidence revealed that circRNAs contribute to immune escape via a circRNA-miRNA-PD-1/PD-L1 axis,^123^ indicating that regulating PD-1/PD-L1 expression by targeting related circRNAs may be an innovative direction of future immune checkpoint therapeutic research for TNBC treatment.

## Discussion

This review summarizes the anticancer efficacy of chemotherapy and targeted therapy (Table 1). The unsatisfactory clinical outcomes of these therapies have underscored the need for the development of novel therapies. This review also discusses the biogenesis and molecular mechanisms of circRNAs. circRNAs have critical roles in TNBC owing to their unique properties. Furthermore, this review summarizes the recent findings on the role of circRNAs in TNBC (Tables 2 and 3), with a special focus on their experimental and clinical values in cancer research. The application of high-throughput screening technology has enabled the identification of dysregulated circRNAs in multiple cancers, including TNBC, indicating circRNAs can serve as potential diagnostic and prognostic biomarkers, therapeutic targets, chemotherapy-related resistance regulators, and therapeutic vectors.

### Current limitations

Researchers have long been challenged by how to promote circRNAs into clinical utilization as noninvasive biomarkers for early detection. Most studies on circRNAs are single-center retrospective studies, limiting the translation of circRNAs from laboratory findings to clinical practice. The clinical application of circRNAs can be achieved through screening and validating circRNAs, as biomarker candidates, in studies comprising a large number of patients. Moreover, the development of bioinformatics methods and standardized techniques for the application of circRNAs can aid in the early diagnosis of cancer and improve the efficacy of cancer treatment. Comprehensive studies on the differentially expressed circRNAs involved in chemoresistance in BC may suggest potential targets for further functional analysis according to circRNA microarray data.^110^ circRNAs are prospectively therapeutic for patients with TNBC by binding oncogenic with antisense oligonucleotides via BSJs or promoting the effect of tumor suppressors. Fortunately, ectogenic artificial circRNAs can be constructed by enzymatic ligation *in vitro* and stably expressed in cancer cells functioning as ceRNAs *in vivo*.^124^, ^125^, ^126^ We believe that synthetic circRNAs could become a promising therapeutic in molecular therapy and medicine.

Second, because the detecting technology has yet to be established, circRNA profiles in TNBC have not been determined. Once the homeostasis of the tumor microenvironment has been broken down, the expression of circRNAs varies. As circRNAs do not have poly(A) tails and 5¢ to 3¢ polarity, they cannot be recognized using poly(A)^+^ RNA-seq. Hence, circRNAs must be identified by modified RNA-seq library preparation protocols, which involve ribosomal RNA depletion, poly(A) depletion, and RNase R treatment (to digest linear RNAs). Although RNase R treatment can enrich circRNAs, it can yield heterogeneous samples.^127^ Additionally, there is no gold-standard algorithm for determining circRNA expression. Hence, there is a need to utilize multiple algorithms for determining circRNA expression. Experimental approaches must be developed to validate the algorithmic prediction of circRNAs, as computational prediction is associated with a high number of false-positive results.^128^ Quantitative real-time PCR can amplify the sequences of cognate linear RNAs identical to those of the BSJ site. However, northern blotting is an efficient method to validate RNA species.^28^ In quantitative real-time PCR, the artifact of back-spliced sequences generated by template switching of the reverse transcriptase and rolling circle amplification can be falsely validated as circRNAs. The use of diverse primers and probes can aid in the identification of both circRNAs and linear RNAs based on the target sequences within circRNA-producing exons. circRNAs exhibit slower migration than those of their linear counterparts in denaturing polyacrylamide gel electrophoresis. Additionally, high-resolution microscopy combined with fluorescence *in situ* hybridization may also aid in the identification and isolation of pure circRNAs^129^ in TNBC. Dedicated microarray platforms are an alternative method to identify circRNAs. The recent development of RNA-seq and microarrays to profile TNBC samples has enabled the rapid detection of corresponding dysregulated circRNAs in TNBC.

Third, the limitation of illustrating the comprehensive mechanism of circRNAs in tumors is troublesome. Dysregulated circRNAs play crucial roles involving tumor metabolism,^130^ targeting mRNA binding,^123^ escape from immune surveillance,^131^ homeostasis of the tumor microenvironment, and chemotherapy resistance,^132^ but explorations of their underlying mechanisms have only skimmed the surface. Notably, the ceRNA hypothesis has not been proved yet. The miRNA sponge effect of circRNAs was inhibited owing to the insufficient abundance of circRNAs compared to that of miRNAs and the deficiency of compromising miRNA activities.^133^ Therefore, considering the quantitative relationship between a miRNA and its target site of circRNA is dazzlingly significant.^134^ The mitigation of dysregulated circRNA expression could be a promising approach for the TNBC therapy. In addition, in the overexpression or depletion of specific circRNAs, it is challenging to obtain gain-of-function or loss-of-function mutants without disrupting the residing genes. Similarly, the strategies for circRNAs knockdown and knockout are tricky. The recently developed RNAi-mediated degradation^135^ and RNA-targeting Cas13 system^136^ are promising tools for disrupting circRNA expression. However, these approaches cannot specifically target circRNAs, which may result in off-target effects. Moreover, strategies used to knock out linear mRNA are not applicable for the knockout of circRNAs. Future studies must combine these knockout technologies with high-throughput screening to facilitate the annotation of circRNA functions in the genome or for CRISPR-Cas9-mediated genome editing. The *trans* overexpression of circRNAs can be achieved using overexpression plasmids. However, the *trans* overexpression of circRNAs from a plasmid construct is accompanied by abundant unprocessed and processed linear RNAs.^92^ Additionally, *cis* overexpression of circRNAs can be specifically achieved by replacing the weak intronic RNA pair with a strong intronic pair.

Fourth, the delivery of circRNAs to the recipient cells is challenging for exerting prolonged therapeutic effects and evading immunological rejection in TNBC. Further studies are needed to elucidate the molecular mechanisms underlying circRNA circularization, localization, and binding in TNBC. The majority of circRNAs accumulated in cytoplasm, while the control process of the localization or nuclear export remained unknown. CircSplice, a new algorithm proposed by Feng et al.,^137^ can detect internal alternative splicing events in circRNAs and identify differential circRNA splicing events under different conditions and hence can be applied to explore the molecular regulatory mechanisms of circRNAs. Moreover, advanced experimental methods have been proposed to elucidate the detailed binding sites of circRNAs involved in their sponging activity, including AGO2 RNA immunoprecipitation (RIP) combined with luciferase reporter assay, RIP followed by sequencing, and RNA pull-down combined with mass spectrometry.^138^^,^^139^ The rapid development of technologies involving extracellular vesicles and nanoparticles may address the limitation associated with circRNA delivery.

### Future perspectives

The recent advances in sequencing techniques have improved our understanding of the regulatory role of circRNAs in TNBCs. However, studies to validate these findings have not progressed, so that more advanced technologies are necessary for circRNA detection, interference, and reintroduction. Notably, Li et al.^140^ revealed that the CRISPR-Cas13 technique is able to knock down circRNAs with impervious effects on homologous mRNAs by targeting sequences spanning BSJ sites via guide RNA featured in circRNAs. Next, the mechanism of ceRNA function in TNBC must be elucidated after specific circRNAs have been verified. Furthermore, the strong correlation between circRNAs and DNA methylation or functional binding proteins has piqued the interest of the scientific community in recent years.^141^^,^^142^ The biogenesis and functions of circRNAs must be further examined. The natural structure of ribosomal binding sites and ORF enables circRNAs to encode specific peptides. Future studies must develop validation methods, including ORF identification *in vitro*, intracellular peptide identification, and the detection of biological phenotypes of circRNAs in TNBC to examine the peptide translation function of circRNAs. In addition, an increasing number of network databases would be powerful tools to apply in circRNA characterization, functional analyses, and investigation of interaction with other molecules.

Current functional and clinical insights address the critical roles of circRNAs in tumor research, but we hope that these accomplishments are just a starting point. A better comprehension of the mechanisms modulating the circRNA fate, the downstream factors of circRNA regulatory networks, and the clinical relevance of circRNAs would enrich our knowledge of circRNA function in cancer biology and the development of circRNA-based diagnosis, prognosis, and therapeutic methods for TNBC.

## Conclusions

circRNAs must be further annotated to elucidate their mechanisms in tumorigenesis and progression of TNBC and to develop personalized treatments. Studies examining the potential of circRNAs as cancer biomarkers and novel therapeutic targets for TNBC are limited. A comprehensive understanding of circRNAs in the nervous system, carcinogenesis, inflammation events, or other pathological processes can aid in further characterization of the functions of circRNAs.
